# Early versus Delayed Decompression for Traumatic Cervical Spinal Cord Injury: Results of the Surgical Timing in Acute Spinal Cord Injury Study (STASCIS)

**DOI:** 10.1371/journal.pone.0032037

**Published:** 2012-02-23

**Authors:** Michael G. Fehlings, Alexander Vaccaro, Jefferson R. Wilson, Anoushka Singh, David W. Cadotte, James S. Harrop, Bizhan Aarabi, Christopher Shaffrey, Marcel Dvorak, Charles Fisher, Paul Arnold, Eric M. Massicotte, Stephen Lewis, Raja Rampersaud

**Affiliations:** 1 Divisions of Neurosurgery and Orthopedic Surgery, Department of Surgery, University of Toronto, Toronto, Ontario, Canada; 2 Division of Spinal Disorders, Department of Neurosurgery and Orthopedic Surgery, Thomas Jefferson University, Philadelphia, Pennsylvania, United States of America; 3 Department of Neurosurgery, University of Maryland, Baltimore, Maryland, United States of America; 4 Departments of Neurosurgery and Orthopedic Surgery, University of Virginia, Charlottesville, Virginia, United States of America; 5 Department of Orthopedic Surgery, University of British Columbia, Vancouver, British Columbia, Canada; 6 Department of Neurosurgery, University of Kansas, Kansas City, Kansas, United States of America; Hertie Institute for Clinical Brain Research, University of Tuebingen, Germany

## Abstract

**Background:**

There is convincing preclinical evidence that early decompression in the setting of spinal cord injury (SCI) improves neurologic outcomes. However, the effect of early surgical decompression in patients with acute SCI remains uncertain. Our objective was to evaluate the relative effectiveness of early (<24 hours after injury) versus late (≥24 hours after injury) decompressive surgery after traumatic cervical SCI.

**Methods:**

We performed a multicenter, international, prospective cohort study (Surgical Timing in Acute Spinal Cord Injury Study: STASCIS) in adults aged 16–80 with cervical SCI. Enrolment occurred between 2002 and 2009 at 6 North American centers. The primary outcome was ordinal change in ASIA Impairment Scale (AIS) grade at 6 months follow-up. Secondary outcomes included assessments of complications rates and mortality.

**Findings:**

A total of 313 patients with acute cervical SCI were enrolled. Of these, 182 underwent early surgery, at a mean of 14.2(±5.4) hours, with the remaining 131 having late surgery, at a mean of 48.3(±29.3) hours. Of the 222 patients with follow-up available at 6 months post injury, 19.8% of patients undergoing early surgery showed a ≥2 grade improvement in AIS compared to 8.8% in the late decompression group (OR = 2.57, 95% CI:1.11,5.97). In the multivariate analysis, adjusted for preoperative neurological status and steroid administration, the odds of at least a 2 grade AIS improvement were 2.8 times higher amongst those who underwent early surgery as compared to those who underwent late surgery (OR = 2.83, 95% CI:1.10,7.28). During the 30 day post injury period, there was 1 mortality in both of the surgical groups. Complications occurred in 24.2% of early surgery patients and 30.5% of late surgery patients (p = 0.21).

**Conclusion:**

Decompression prior to 24 hours after SCI can be performed safely and is associated with improved neurologic outcome, defined as at least a 2 grade AIS improvement at 6 months follow-up.

## Introduction

The prevalence of traumatic spinal cord injury (SCI) worldwide is approximately 750 per million with an annual incidence that appears to be rising [1]. Given the impact of SCI on the individual and society, it is clear that effective therapies aimed at reducing the extent of tissue destruction and improving neurologic outcomes after the initial spinal cord trauma are urgently needed. Current concepts of the pathophysiology of acute SCI indicate that there are both primary and secondary mechanisms that lead to neurologic injury [2], . The primary injury, usually caused by rapid spinal cord compression and contusion, initiates a signaling cascade of down-stream events collectively known as secondary injury. Preventing and mitigating these secondary mechanisms is where opportunity for neuroprotection lies and where most attempts at therapeutic intervention have been staged.

The balance of existing laboratory evidence supports the theory that decompressive surgery of the spinal cord after SCI attenuates secondary injury mechanisms and improves neurological outcomes [5], [6], [7], [8], [9], [10], [11], [12], [13], [14], [15]. Furthermore, the strength of this neuroprotective effect seems to vary inversely with the time elapsed from injury to the decompression [8], [15]. This work has translated into the clinical hypothesis that those who undergo surgery in a timely fashion post injury will experience less neural tissue destruction and improved clinical outcomes as compared to injury matched patients treated conservatively or with surgery in a delayed fashion.

However, the clinical evidence compiled to date has failed to provide robust support for this hypothesis. One small randomized controlled trial and several other prospective studies showed no benefit to early decompression, with the caveat that early was defined as within 72 hours from the time of injury and that enrolment was limited to a single centre [16], [17], [18], [19]. In contrast, a systematic review suggested that decompression within 24 hours resulted in improved outcomes compared to both delayed decompression and conservative treatment [20]. Based on the best available evidence, the Spine Trauma Study Group adopted the 24 hour cutoff to define early versus late decompressive surgery after SCI [21].

To date, there have been no published studies that have systematically examined a large cohort of patients who underwent decompression earlier than 24 hours. To address this void, we present the results of the Surgical Timing in Acute Spinal Cord Injury Study (STASCIS), a multi-center prospective cohort study that was undertaken to compare the relative effectiveness of early (less than 24 hours post injury) versus late (24 hours or greater post injury) surgery with respect to neurological outcome 6 months post cervical SCI. As secondary questions, we assessed the impact of surgical timing on in-hospital postoperative complication rates and mortality.

## Methods

We have completed a prospective, multicenter, cohort study involving hospitals at 6 institutions throughout North America: 1) University of Toronto, Toronto, Ontario, Canada 2) Thomas Jefferson University, Philadelphia, PN, USA 3) University of Virginia, Charlottesville, VA, USA 4) University of Maryland, Baltimore, MD, USA 5) University of British Columbia, Vancouver, British Columbia, Canada; 6) University of Kansas, Kansas City, KS, USA. Each of the hospitals involved are specialized in the management of spinal trauma and spinal cord injury. Patient enrollment began in August 2002 and ended in September 2009. Research ethics board approval was obtained at each of the 6 centers prior to beginning enrollment. During this period any SCI patient presenting to one of these institutions was assessed for suitability against a predefined set of inclusion and exclusion criteria (Table 1).

**Table 1:** Inclusion/Exclusion Criteria.

Inclusion Criteria	Exclusion Criteria
1) Male or female	1) Cognitive impairment preventing accurate neurologic assessment
2) Ages 16–80	2) Penetrating injuries to the neck
3) Initial GCS >13	3) Pregnant females
4) Initial AIS grade A–D	4) Pre-injury major neurologic deficits or disease (i.e. ischemic stroke, Parkinson's Disease)
5) Cervical spinal cord compression confirmed by MRI or CT Myelography	5) Life threatening injuries which prevent early decompression of the spinal cord
6) Patient or Proxy willing to provide consent for enrollment	6) Arrival at health center >24 hours after SCI
7) Neurological Level of Injury between C2 and T1	7) Surgery >7 days after SCI

At presentation, neurologic examination was performed as per standards established by the American Spinal Injury Association (ASIA) and injury characteristics were classified according to neurologic level of injury (NLI), ASIA motor score (AMS), ASIA sensory score (ASS) and the overall ASIA Impairment Scale (AIS) grade. The baseline ASIA assessment was performed within 24 hours on all subjects. The primary outcome measure of interest was ordinal change in AIS grade at 6-months follow-up. The 6 month time period for follow-up was based on recommendations used in the NASCIS and Sygen trials as well as on the findings of previous natural history studies which demonstrate that the vast majority of neurological recovery occurs during this period [22], [23], [24], [25], [26], [27]. Additional clinical parameters collected at admission included patient age, gender, mechanism of injury, Charleson Co-morbidity Index (CCI) and initial Glasgow Coma Scale (GCS) score. Prior to study enrollment, each patient underwent a plain X-Ray, computed tomographic (CT) scan and magnetic resonance imaging (MRI) study of their cervical spine. Particular note was made of the presence of spinal cord compression on MRI as this defined one of the major study inclusion criteria. Spinal cord compression was defined by the method we have previously described [28]. For patients unable to undergo MRI, CT myelography was performed.

After initial clinical and radiographic evaluation, study eligibility was determined. After enrollment, subjects underwent either early (<24 hours after injury) or late (≥24 hours after injury) decompressive surgery of the cervical spinal cord. Decision of surgical timing was dependent on the time elapsed post injury at patients' hospital arrival, the time required to obtain diagnostic investigations, and the discretion of the attending spinal surgeon. The specifics of the surgical intervention, such as the direction of approach (anterior vs. posterior) and number of levels decompressed, were also decided based on the judgment of the attending spinal surgeon. In all cases, decompression was accompanied by an instrumented fusion procedure. Apart from the surgical management, all patients received appropriate medical support according to the 2002 American Association of Neurological Surgeons cervical SCI guidelines, which included permissive or induced hypertensive therapy (mean BP >85 mm Hg) [29], [30], [31]. Methylprednisolone was used as per the discretion of the treating team according to the recommendations of the NASCIS-2 study [25]. CT imaging was performed within 72 of surgery for all patients, and read by a site specific radiologist, to establish the patency of the spinal canal in the postoperative setting. In specific circumstances, such as postoperative neurological deterioration, repeat MRI scan was performed to evaluate the spinal cord and to exclude the presence of ongoing spinal cord compression. Lastly, all patients underwent a post-operative rehabilitation regimen, tailored to individual and injury specific factors.

When unilateral or bilateral cervical facet dislocation was diagnosed on initial X-ray or CT scan, these patients were reduced, on an emergent basis, by either closed or open means. A MRI was obtained following closed reduction to document the degree of decompression of the spinal cord achieved. If the post reduction MRI demonstrated complete resolution of spinal cord compression, then the time at which closed reduction was achieved was recorded as the time of decompression.

After surgery, patients were analyzed in groups according to the timing of their operative intervention. A trained research assistant, blinded to the timing of patients' surgical treatment, performed follow-up neurological examinations at acute hospital discharge and 6 months post-operatively. Documentation of relevant in-patient postoperative complications was also performed. For the complications analysis, patients without 6 month follow-up data were also included since complications data from the acute hospital admission were available for all patients enrolled.

### Statistical Analysis

All analyses were performed using SAS 9.2. To determine the effects of surgical timing on AIS grade improvement and to account for baseline discrepancies between the cohorts, we performed a generalized ordinal logistic regression analysis. The dependent variable was ordinal change in AIS grade from pre-operative baseline to 6 months post-operatively, and the independent variable of interest was defined as surgical timing (early vs. late). Predictor variables related to baseline patient characteristics, such as age, gender, complete (AIS A) vs. incomplete (AIS B–D) neurological status at admission and steroid administration, were included in the initial model and sequentially eliminated in a backwards fashion, if their corresponding p-value was greater than 0.05. Continuous variables were compared between the treatment groups using the student t-test. Categorical data were analyzed by Fisher's exact and chi-squared tests.

## Results

### Study Population

**Figure 1:**
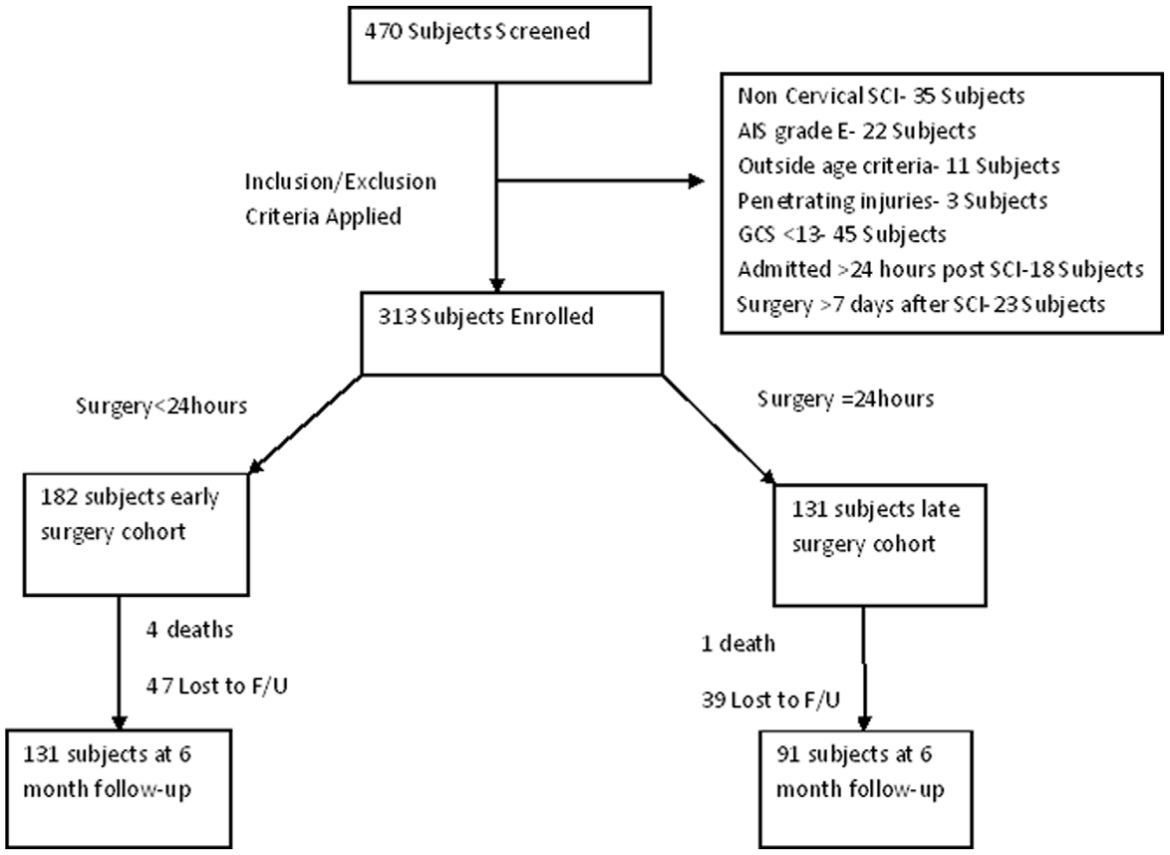
Patient Flow.

A total of 470 subjects were screened for enrollment of which 313 satisfied study inclusion and exclusion criteria (Figure 1). Of the 313 study participants, 182 underwent surgery less than 24 hours after SCI and were considered the early surgery cohort. The remaining 131 patients underwent surgery at or after 24 hours post SCI and were considered the late surgery cohort. Both groups were followed prospectively over time until 6 months post injury. During the study period, 5 patients died and 86 patients were lost to follow-up, leaving a total study population of 222 on which to base the 6 month analysis. In the early surgery group, 4 patients died and 47 were lost to follow-up, leaving 131 patients. In the late surgery group 1 patient died and 39 were lost to follow-up, leaving 91 patients. Within the early surgery group the mean time to surgery was 14.2(±5.4) hours and 48.3(±29.3) hours within the late surgery group (p<0.01). No patient in either group underwent repeat operation for inadequate decompression as determined by postoperative imaging.

Table 2 gives a comparative breakdown of the demographic and injury characteristics of the entire study population, the early surgery group and the late surgery group. In the early surgery cohort the mean age was 45.0±17.2 with 140 males (76.9%) and 42 females (23.1%). In the late surgery cohort the mean age was 50.7±15.9 years with 96 males (73.3%) and 35 females (26.7%). There was no significant difference in the distribution of gender between the two groups, however there was a statistically significant difference in mean age between the groups, with patients in the early surgery cohort tending to be younger (p<0.01). The neurologic status on admission was significantly different between the cohorts with AIS grade A's and B's overrepresented in the early group and C's and D's more common in the late group (p<0.01). The majority of injuries in both cohorts resulted from either motor vehicle accidents or falls with no significant differences in etiology between groups.

**Table 2:** Patient Demographics and Injury Characteristics.

characteristics	Overall N = 313	Early surgery N = 182	Late Surgery N = 131	P value
**mean age ± SD**				**P<0.01**
	47.4±16.9	45.0±17.2	50.7±15.9	
**Gender n(%)**				**p>0.05**
Male	236 (75.4%)	140 (76.9%)	96 (73.3%)	
Female	77 (24.6%)	42 (23.1%)	35 (26.7%)	
**Etiology**				**p>0.05**
Motor Vehicle Accident	119 (38.0%)	76 (41.8%)	43 (32.8%)	
Fall	121 (38.7%)	64(35.1%)	57 (43.5%)	
assault – blunt	13 (4.2%)	8 (4.4%)	5 (3.8%)	
Sports	3 (9.6%)	16 (8.8%)	12 (9.2%)	
Other	3 (9.6%)	18 (9.9%)	14 (10.7%)	
**Baseline ASIA Impairment Scale grade**				**P<0.01**
A	101(32.3%)	65 (35.7%)	36 (27.5%)	
B	54 (17.3%)	40 (22.0%)	14 (10.7%)	
C	66 (21.1%)	32 (17.6%)	34 (26.0%)	
D	92 (29.4%)	45 (24.7%)	47 (35.9%)	
**Charleson Co-morbidity index ≥1**				**p>0.05**
	74(23.6%)	40(22.0%)	30(26.0%)	
**Glasgow Coma Scale ± SD**				**P>0.05**
	14.9±0.4	14.9±0.4	14.9±0.4	

In the entire study population 194 patients (62.0%) received steroids at hospital admission, with a significantly higher proportion of administration in the early as compared to the late group(p = 0.04).

### Neurologic Recovery at 6 months

**Figure 2:**
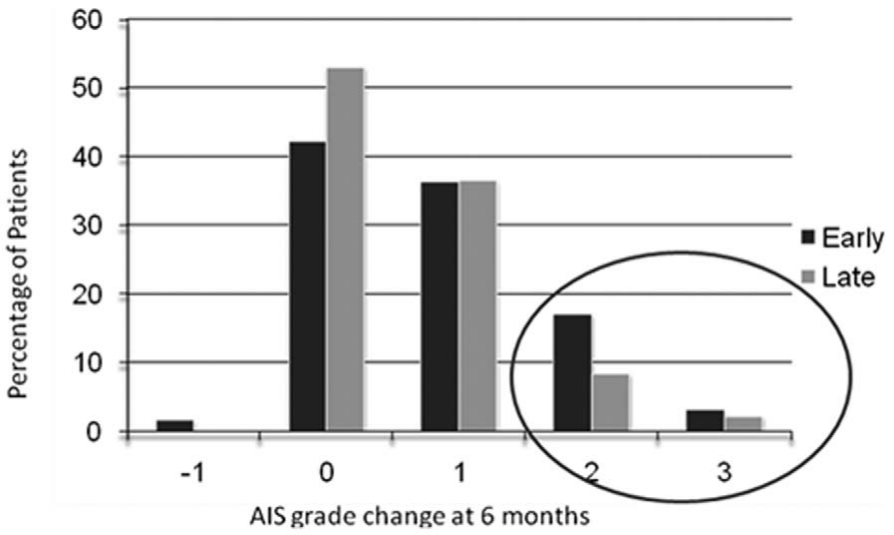
AIS Grade Improvement at 6 months: Early vs. Late Surgery.

In the entire study group, the degree of neurologic improvement was significant as measured by change in AIS grade from presentation to 6 months follow-up (p = 0.02) (Table 3). In the early surgery group, AIS grade improvement was as follows: 56 (42.7%) had no improvement, 48 (36.6%) had a 1 grade improvement, 22 (16.8%) had a 2 grade improvement, 4 (3.1%) had a 3 grade improvement and 1 (0.8%) had a 1 grade worsening (Table 4). In the late group, AIS grade improvement was as follows: 46 (50.6%) had no improvement, 37 (40.7%) had a 1 grade improvement, 8 (8.8%) had a 2 grade improvement, and no patient worsened (Table 5). Based on this information, 74 patients (56.5%) in the early group and 45 patients (49.5%) in the late group experienced at least a 1 grade improvement (*early vs. late surgery:* OR = 1.33, 95% CI:0.78,2.27) and 26 patients (19.8%) in the early group and 8 patients (8.8%) in the late group experienced at least a 2 grade improvement (*early vs. late surgery:* OR = 2.57, 95% CI:1.11,5.97) at 6 months (Figure 2).

**Table 3:** Ordinal changes in AIS grade from pre-op to 6 months follow-up: Total Study Population.

Preoperative AIS grade	A	B	C	D	E	Total
**A**	42	18	9	2	0	71
**B**	1	11	11	17	2	42
**C**	0	0	7	32	4	43
**D**	0	0	0	42	24	66

**Table 4:** Ordinal changes in AIS grade from pre-op to 6 months follow-up: Early Surgery group.

Preoperative AIS grade	A	B	C	D	E	Total
**A**	25	11	6	2	0	44
**B**	1	7	9	12	2	31
**C**	0	0	2	16	4	22
**D**	0	0	0	22	12	34

**Table 5:** Ordinal changes in AIS grade from pre-op to 6 months follow-up: Late Surgery group.

Preoperative AIS grade	A	B	C	D	E	Total
**A**	17	7	3	0	0	27
**B**	0	4	2	5	0	11
**C**	0	0	5	16	0	21
**D**	0	0	0	20	12	32

In development of the multivariate regression model, after backwards elimination of predictors with p-values >0.05, in addition to surgical timing, only complete vs. incomplete status and steroid administration remained in the regression equation (Table 6). The odds of at least a 2 grade AIS improvement were 2.8 times higher amongst those who underwent early surgery as compared to those who underwent late surgery, after adjusting for preoperative neurologic status and steroid administration (OR = 2.83, 95% CI:1.10,7.28). The odds of a 1 grade AIS improvement were 1.4 times higher amongst those who underwent early surgery as compared to those who underwent late surgery, after adjusting for preoperative neurologic status and steroid administration, however this was not statistically significant (OR = 1.37, 95% CI:0.80,2.57).

**Table 6:** Results of generalized ordinal logistic regression model assessing the effect of early vs. late surgical decompression, adjusted for preoperative neurological status and steroid administration.

Predictor Variable	Odds Ratio with 95% CI	p-value
Early vs. Late surgery≥2 grade AIS improvement	2.83 (1.10,7.28)	P = 0.03
Early vs. Late surgery1 grade AIS improvement	1.38 (0.74, 2.57)	P = 0.31

### Postoperative Complications and Mortality

Of the 313 patients who were enrolled and underwent surgery, there were a total of 97 major post-operative inpatient complications that occurred in 84 individuals. Table 7 provides a synopsis of the postoperative complications in each group. In the early group, 44 individuals (24.2%) experienced 48 complications and, in the late group, 40 individuals (30.5%) experienced 49 complications. Although there was a lower proportion of patients in the early surgical group who experienced at least one complication (24.2%) as compared to the late surgery group (30.5%), this difference was not statistically significant (p = 0.21).

**Table 7:** Inpatient Postoperative Complications.

Complication	Total Population	Early Surgery	Late Surgery
**Cardiopulmonary**	66 (68.0%)	32 (66.7%)	34 (69.4%)
**Construct Failure Requiring Surgery**	4 (4.1%)	3 (6.3%)	1 (2.0%)
**Deep Wound Infection**	2 (2.1%)	0	2 (4.1%)
**Neurologic Deterioration**	5 (5.2%)	4 (8.3%)	1 (2.0%)
**Pulmonary Embolism**	4 (4.1%)	2 (4.2%)	2 (4.1%)
**Systemic Infection**	14 (14.4%)	6 (12.5%)	8 (16.3%)
**Wound Dehiscence**	1 (1.0%)	1 (2.1%)	1 (2.0%)
**Totals**	**97**	**48**	**49**

During the 30 day post injury period there was 1 mortality in both the early and late surgery groups. The death in the early surgery patient was secondary to a postoperative myocardial infarction and the death in the late surgery patient was related to pulmonary complications. Subsequent to the 30 day post injury time window, 3 deaths occurred in the early surgery group, all from cardio-respiratory causes, and no deaths occurred in the late surgery group.

## Discussion

STASCIS represents the largest prospective multi-center study comparing early vs. late surgical decompression in the setting of acute traumatic spinal cord injury. Results of the unadjusted analysis indicate a significant difference, favoring the early group, in the proportion of patients recovering at least 2 AIS grades at 6-months follow-up. The Sygen trial, the largest therapeutic trial in SCI, defined significant neurologic recovery as at least a 2 grade AIS improvement at 6 months follow-up [22]. In applying a similar definition to the current study, the unadjusted analysis demonstrated a more favorable neurologic recovery amongst those treated with early surgery. The multivariate regression analysis, adjusted for preoperative neurological status and steroid administration, continued to demonstrate that patients who underwent early surgery were more likely to improve at least 2 AIS grades at follow-up.

Having demonstrated the potential for improved neurological outcomes with early surgical decompression, the obvious question becomes: how does one define ‘early’? The notion of early surgery stems from an increased understanding of secondary mechanisms of SCI deduced primarily from animal data [32], [33]. In a recent systematic review of the preclinical literature, 19 studies were identified evaluating decompression in several different animal SCI models [34]. Of these, 11 reported a time dependent effect favoring early surgery, with outcome variably defined in terms of follow-up functional status, degree of tissue destruction on post-mortem histological analysis or electrophysiological findings. In most of these animal studies, the timing of surgical decompression was in the range of 8 to 24 hours post injury, an experimental model that is difficult to replicate in clinical situations where practical factors limit this possibility. As a result, while the preclinical literature establishes a clear biologic rationale to support early decompressive surgery, it is impossible to extract from these studies an optimal therapeutic window for the surgical treatment of human SCI patients. With respect to the existing clinical evidence, a recent systematic review of the human literature concluded that decompression within 24 hours of injury resulted in improved outcomes compared to either delayed surgery (>24 hours) or conservative treatment [20]. To elaborate, the SCI literature has been historically variable on the definition of timing. Out of 22 studies attempting to define optimal timing for surgery after acute traumatic SCI, 9 utilized the 24 hour limit to define an early decompressive operation [35], [36], [37], [38], [39], [40], [41], [42], [43], 8 used 72 hours [18], [19], [44], [45], [46], [47], [48], [49], and 4 used other benchmarks such 8 hours, 48 hours or 4 days [50], [51], [52], [53]. Importantly, no study has associated adverse neurologic outcomes with early surgical intervention, regardless of a specific time cutoff. Based on the biology of secondary mechanisms in spinal cord injury, the Spine Trauma Study Group [21] has operationally defined early intervention as occurring within 24 hours. Our decision to employ the 24 hour definition was based on analyzing the available preclinical and clinical data which suggested that outcomes, neurologic and otherwise, would be potentially optimized if surgery was performed between 8 and 24 hours post injury. In spite of this, all recommendations made to date have lacked the support of a large systematic comparative analysis evaluating the relative efficacy of various surgical timing cutoff points.

Comparing the rates of AIS grade conversion in the current study to those reported in other large SCI series, it is clear that we report superior rates of recovery, particularly amongst AIS grade A patients, regardless of the surgical group considered. When both cohorts are taken together, 40% of preoperative AIS grade A patients (43% in the early group and 37% in the late group) experienced at least a 1 grade improvement, compared to historical rates of 15–25% [54]. We attribute this difference to our exclusion of patients with severe concomitant injuries, use of a rigorous, standardized protocol of management including induced hypertensive therapy, and focus on a cervical cohort, where the potential for recovery is greater than for those with severe thoracic injuries.

The pivotal point of this study was to compare the relative effectiveness of early and late surgical decompression with respect to neurological outcomes for those sustaining traumatic cervical SCI. As with any methodological design, there are certain limitations that are recognized. Although a randomized trial would have been, in theory, methodologically superior to address the therapeutic efficacy of this intervention, we chose a prospective cohort design for both practical and ethical reasons. From a practical standpoint, it has been shown in previous feasibility studies that between 23.5% and 51.4% of SCI patients can undergo an operation within the first 24 hours after injury due mainly to transport and life saving measures [35], [43]. If we were to perform a study randomizing patients to early versus late decompression, the study population would be based only on the one quarter to one half of the total SCI population who are eligible to undergo surgery within 24 hours of injury, introducing significant selection bias. From an ethical standpoint, there was consensus among participating surgeons that it would be unacceptable to withhold decompressive surgery to a patient deteriorating neurologically with significant concomitant spinal cord compression; highlighting the point that it is nearly impossible to achieve clinical equipoise in a trauma population, a prerequisite for a proper randomized trial.

In the current study, all patients, regardless of whether they received early or late surgery, underwent a standard ASIA neurological examination within 24 hours of injury. Results of neurological examinations performed within this period have shown to be valid and are consistent with examination results obtained at 72 hour post injury, except amongst patients with an associated traumatic brain injury [55], [56]. In order to ensure that initial neurological assessments were not confounded by extraneous factors, patients with head injuries (GCS ≤13) and significant poly-trauma were not enrolled. Another perceived threat to the validity of the acute neurological assessment has previously been the presence of spinal shock. However, according to the most recent evidence on the topic, spinal shock likely represents an ongoing physiologic continuum consisting of 4 stages, occurring in virtually all patients with severe SCI, beginning within minutes after injury and continuing for up to 12 months [57]. Based on this modern definition, it would be inappropriate to identify an SCI patient as being “in” or “out of” spinal shock for purposes of classification within a study.

### Study Limitations

The early surgery group included patients with a slightly lower mean age and contained a significantly greater proportion of patients with a more severe degree of initial injury as compared to the late group. These discrepancies may be a reflection of study surgeons tending to be more aggressive in the treatment of younger SCI patients with a more severe injury. An alternative explanation might be that younger patients generally have fewer co-morbidities and are less complicated to resuscitate enabling an expeditious path to decompression. Nonetheless, the multivariate analysis which controlled for baseline differences between the groups, confirmed that early decompression within 24 hours of acute cervical SCI was associated with improved neurologic outcomes. We also recognize that a fraction of the study population (27%) was lost to long term review, although our follow-up rates compare favorably to other major prospective studies in SCI including NASCIS I where the loss to follow-up at 6 months was 31% [24]. This is attributed to the inherent challenges of following a large group of trauma patients, many of whom reside in rural communities separated by large distances from the specialized study centers.

### Conclusion

In the current study, decompressive surgery prior to 24 hours after SCI was performed safely and was associated with improved neurologic outcome defined as at least a 2 grade AIS improvement at 6 months follow-up. Of note, the results of this study appear to validate a growing consensus among spine surgeons favoring early surgical intervention for SCI [21]_ENREF_21. However, these conclusions must be tempered given the inherent limitations of the cohort study design used in the STASCIS study. Therefore, further study is necessary to more accurately define which SCI patients benefit the most from early surgical intervention.
